# Successful salvage surgery followed by second ALK–TKI after alectinib failure in a patient with ALK-positive NSCLC

**DOI:** 10.1186/s40792-022-01408-7

**Published:** 2022-04-02

**Authors:** Hiroshi Hashimoto, Kazuyuki Komori, Koji Kameda, Shinichi Taguchi, Yuichi Ozeki

**Affiliations:** 1 Department of Thoracic Surgery, National Defense Medical College, 3-2, Namiki, Tokorozawa, Saitama 359-8513 Japan; 2 Department of Thoracic Surgery, Tokorozawa Meisei Hospital, 5095, Yamaguchi, Tokorozawa, Saitama 359-1145 Japan

**Keywords:** Anaplastic lymphoma kinase, Tyrosine kinase inhibitor, Lung cancer, Salvage surgery, Alectinib, Lorlatinib

## Abstract

**Background:**

Anaplastic lymphoma kinase (ALK)–tyrosine kinase inhibitors (TKIs) have been approved for the therapy of locally advanced non-small cell lung cancer (NSCLC) caused by ALK rearrangement. However, its treatment after failure of initial ALK–TKI therapy remains controversial.

**Case presentation:**

A 47-year-old woman with a hemosputum was diagnosed with adenocarcinoma of the left lung (cT2bN3M0, stage IIIB). Gene mutation analysis indicated positive ALK translocation. Alectinib was selected as the first-line treatment. Although the treatment effect was determined as a partial response, the main tumor regrew. Alectinib was discontinued, and salvage surgery was performed without causing morbidity. The pathological diagnosis was pleomorphic carcinoma without lymph node metastasis (yp-T2bN0). After surgery, lorlatinib was administered as the second-line treatment for 8 months until the patient could not tolerate continuation. Computed tomography scan revealed no lung cancer recurrence 14 months after discontinuation.

**Conclusions:**

Our experience with this case suggests that salvage surgery after alectinib treatment followed by lorlatinib therapy may be effective for initially unresectable ALK-positive NSCLC.

## Background

Although anaplastic lymphoma kinase (ALK)–tyrosine kinase inhibitors (TKIs) have been approved for the treatment of locally advanced non-small cell lung cancer (NSCLC) caused by ALK rearrangement [1], its treatment after failure of initial ALK–TKI therapy remains controversial.

Here, we report the case of a patient who had ALK-positive locally advanced lung cancer with multiple lymph node metastases and was successfully treated with salvage surgery between the ALK–TKI sequential therapy.

## Case presentation

**Fig. 1:**
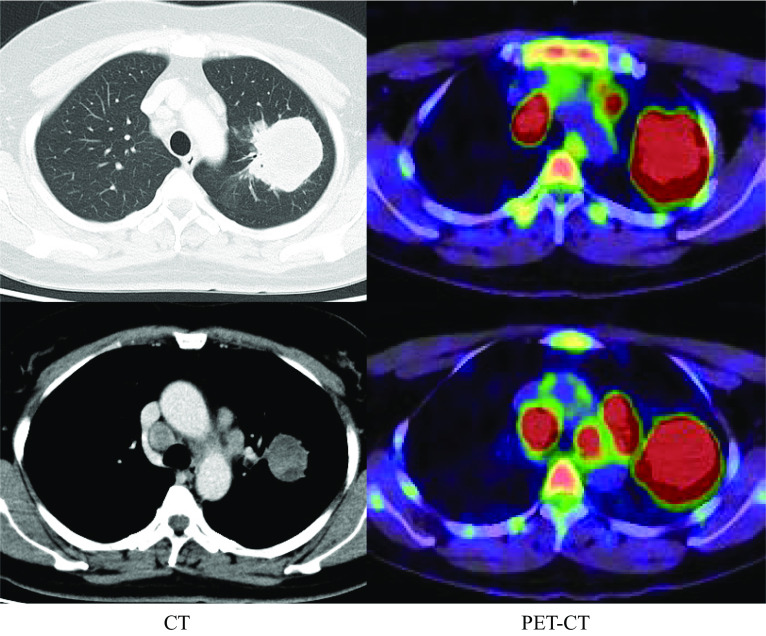
Chest computed tomography (CT) and positron emission tomography (PET) suggest a primary lung tumor in the left upper lobe with mediastinal, left subclavian, and right hilar lymph node metastasis

**Fig. 2:**
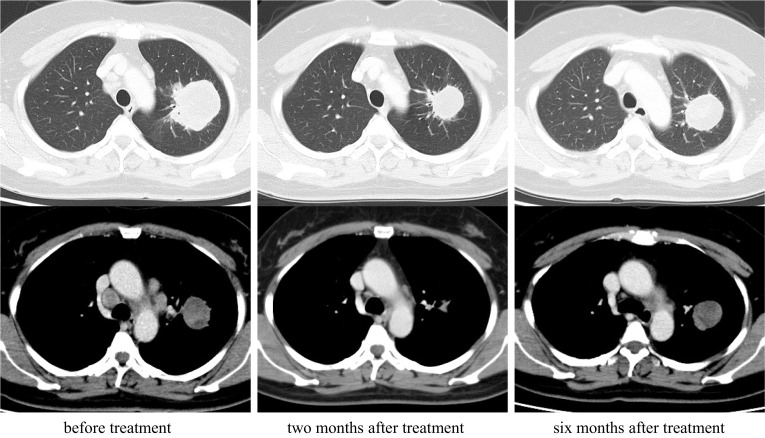
Computed tomography showed that the lung tumor had regrown, while the lymph nodes had shrunk after 6 months of the alectinib treatment

**Fig. 3:**
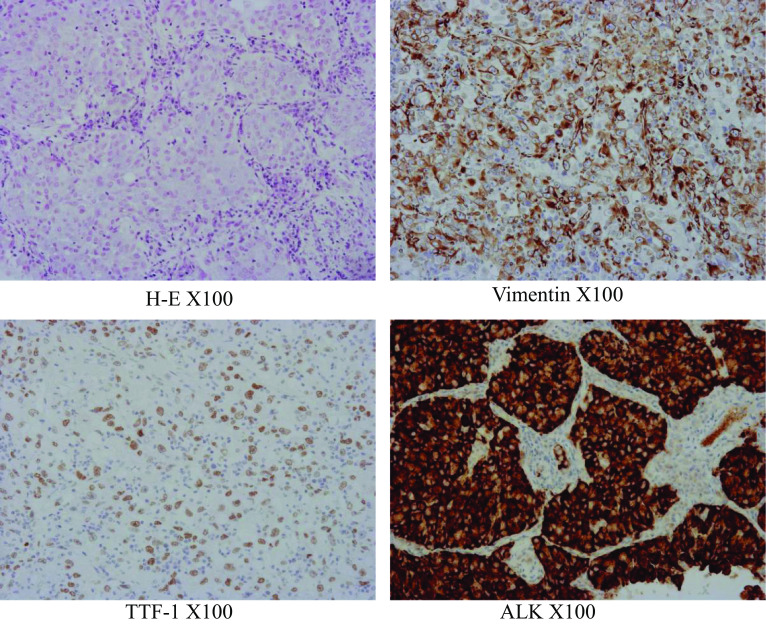
Microscopic findings of the tumor indicated pleomorphic carcinoma with adenocarcinoma features. **a** Hematoxylin and eosin staining (× 200). **b** TTF-1 immunostaining. **c** Vimentin immunostaining. **d** ALK immunostaining. *TTF-1* thyroid transcription factor-1, *ALK* anaplastic lymphoma kinase

A 47-year-old woman, who was a current smoker (1.25 pack-years), was referred to our hospital with a long-term cough followed by hemosputum. Chest radiography revealed a large mass in the upper lung field. Computed tomography (CT) and positron emission tomography findings were suggestive of lung cancer with mediastinal, left subclavian, and right hilar lymph node metastases (Fig. 1). No evidence of distant metastasis, including to the brain, was observed. Tumor marker levels were not elevated (carcinoembryonic antigen: 1.9 ng/ml, cytokeratin 19 fragment: 3.3 ng/ml, and progastrin-releasing peptide: 21.2 pg/ml), whereas serum p53 antibody level was elevated (183 U/ml). She underwent CT-guided percutaneous core-needle biopsy of the left upper lobe tumor. Hematoxylin and eosin staining revealed adenocarcinoma cells with positive immunostaining for thyroid transcription factor-1. Gene mutation analysis showed negative epidermal growth factor receptor (EGFR) mutations, c-ros oncogene-1 rearrangement, and v-Raf murine sarcoma viral oncogene homolog B1 mutations. Tumor cells were positive for staining with the ALK antibody, as seen during immunohistochemical analysis (ALK iScore 5 by iAEP immunohistochemistry). ALK break apart fluorescence in situ hybridization verified the occurrence of ALK rearrangement with a rearrangement-positive cell rate of 76.0%. The patient was treated with alectinib, an ALK–TKI. CT scan showed a marked improvement in both the main tumor and metastatic lymph nodes 2 months after the treatment. However, the lung tumor regrew, while the lymph nodes continued to shrink after 6 months of treatment (Fig. 2). Salvage surgery was planned, and left upper lobectomy with mediastinal lymph node dissection was conducted. The hilar region could not be easily expanded due to the presence of bulky mass and strong adhesion around pulmonary artery branch A3 because of prior alectinib treatment; nevertheless, the operation was completed safely. Her postoperative course was uneventful. The pathological diagnosis of the surgical specimen was pleomorphic carcinoma with adenocarcinoma features without any lymph node metastases (ypT3N0M0, stage IIB; Fig. 3). Approximately 50% residual viable tumor cells were found in the primary lesion site (Ef Ib). After salvage surgery, lorlatinib was administered as a second-line ALK–TKI for 8 months until the patient could not tolerate continued therapy because of adverse effects. CT scan revealed no lung cancer recurrence 14 months after discontinuation.

## Discussion

ALK rearrangement is detected in 2–5% of NSCLC patients [2]. Crizotinib, an ALK–TKI, has shown clinical efficacy in the treatment of NSCLC with ALK rearrangement [1]. However, most patients develop resistance to crizotinib within 1 year of therapy. Second-generation ALK inhibitors such as alectinib and brigatinib have been developed to overcome resistance to crizotinib. Treatment with these second-generation ALK inhibitors has been well tolerated and has shown efficacy in crizotinib-resistant lung cancers with ALK rearrangement [3]. These ALK inhibitors can also be used as first-line treatment for NSCLC with ALK rearrangement [4].

Lorlatinib, a third-generation ALK inhibitor, has the broadest coverage of ALK resistance mutations that have been identified. It has been a standard treatment option for ALK-positive patients who have experienced failure of one or more ALK inhibitors [5].

Although the mechanisms of acquired resistance to initial ALK–TKIs have been elucidated, treatment methods after failure of initial ALK–TKI therapy remain controversial. Some of the treatment options after initial ALK–TKI treatment failure include ALK sequential therapy (switching to lorlatinib or brigatinib) and cytotoxic chemotherapy with or without immune checkpoint inhibitors.

Salvage surgery has been recognized as a surgical treatment for patients with residual tumors or recurring tumors even after definitive chemotherapy and/or radiation therapy.

Shimizu et al. [6] reported that salvage surgery for NSCLC patients after chemoradiotherapy and conventional radiotherapy can cause minimal complications and reasonable outcomes in selected patients. Their patients’ 3-year overall survival (OS) is 67.3%, and the overall 30- and 90-day mortality are 0 and 0.9%, respectively [6]. Although salvage surgery is a feasible therapeutic modality for advanced NSCLC, its survival benefits remain unclear. To date, some salvage surgery trials have been attempted. Ohtaki et al. [7] reported that salvage surgery after TKI treatment may be beneficial for NSCLC patients. They included 36 patients who underwent TKI treatment followed by salvage surgery [7]. They obtained grade 3 adverse events, 3 year OS, and 90 day mortality of 5.6, 75.1, and 0%, respectively. However, many of these patients’ driver gene alterations were *EGFR* mutations. Only three patients had ALK rearrangement.

We have done research on PubMed and also looked up on the internet using lung cancer, ALK, and salvage surgery as key words. Several reports have also described salvage surgery for initially unresectable NSCLC treated with ALK–TKIs [8, 9]. To the best of our knowledge, this report is the first to indicate that salvage surgery after first-line ALK–TKI treatment and subsequent switching to second-line ALK–TKI is effective in a patient with ALK-rearranged locally advanced pleomorphic carcinoma of the lungs. Our patient was first treated with alectinib for 6 months. The lung tumor regrew, while the metastatic lymph nodes exhibited remarkable improvement. The therapeutic response showed a dissociation between the main lung tumor and the metastatic lymph nodes. Salvage surgery was planned, and left upper lobectomy with mediastinal lymph node dissection was performed. Subsequently, second-line ALK–TKI lorlatinib was administered for 8 months, and no evidence of recurrence was found 22 months after salvage surgery. Ohtaki et al. [7] showed the possible benefits of salvage surgery after TKI treatment; however, recurrence-free survival after surgery was only 22%. Such findings suggested that initially unresectable mutation-positive NSCLCs were still systemic diseases even after salvage surgery and postoperative systemic therapy was important for managing the patients. Some postoperative treatment options include restarting initial TKI treatment, using other TKIs, and administering cytotoxic chemotherapy. In our case, lorlatinib was selected as postoperative therapy because of its efficacy after failure of a first-line, second-generation ALK–TKI.

Our case suggested that salvage surgery after first-line ALK–TKI treatment followed by second-line ALK–TKI provides a favorable clinical benefit to the treatment of patients with ALK-rearranged locally advanced NSCLC.

## Conclusions

Our experience with this case suggests that salvage surgery after alectinib treatment followed by lorlatinib therapy may be effective for initially unresectable ALK-positive NSCLC.

## Abbreviations

ALK: Anaplastic lymphoma kinase
TKI: Tyrosine kinase inhibitor
NCSLC: Non-small cell lung cancer
CT: Computed tomography
TTF-1: Thyroid transcription factor-1
EGFR: Epidermal growth factor receptor
OS: Overall survival

## References

[1] Kwak EL, Bang Y, Camidge DR, Shaw AT, Solomon B, Maki RG (2010). First-Line crizotinib versus chemotherapy in ALK-positive lung cancer. N Engl J Med.

[2] Serizawa M, Koh Y, Kenmotsu H, Isaka M, Murakami H, Akamatsu H (2014). Assessment of mutational profile of Japanese lung adenocarcinoma patients by multitarget assays: a prospective, single-institute study. Cancer.

[3] Shaw AT, Gandhi L, Gadgeel S, Riely GJ, Cetnar J, West H (2016). Alectinib in ALK-positive, crizotinib-resistant, non-small-cell lung cancer: a single-group, multicentre, phase 2 trial. Lancet Oncol.

[4] Peters S, Camidge DR, Shaw AT, Gadgeel S, Ahn JS, Kim DW (2017). Alectinib versus crizotinib in untreated ALK-positive non-small-cell lung cancer. N Engl J Med.

[5] Solomon BJ, Besse B, Bauer TM, Felip E, Soo RA, Camidge DR (2018). Lorlatinib in patients with ALK-positive non-small-cell lung cancer: results from a global phase 2 study. Lancet Oncol.

[6] Shimizu K, Ohtaki Y, Suzuki K, Date H, Yamashita M, Iizasa T (2021). Salvage surgery for non-small cell lung cancer after definitive radiotherapy. Ann Thorac Surg.

[7] Ohtaki Y, Shimizu K, Suzuki H, Suzuki K, Tsuboi M, Mitsudomi T (2021). Salvage surgery for non-small cell lung cancer after tyrosine kinase inhibitor treatment. Lung Cancer.

[8] Horio Y, Mizuno T, Sakao Y, Inaba Y, Yatabe Y, Hida T (2019). Successful salvage surgery following multimodal therapy in a patient who harboured ALK-rearranged advanced lung adenocarcinoma with multiple organ metastases. Respirol Case Rep.

[9] Iwamoto N, Matsuura Y, Ninomiya H, Uchibori K, Yanagitani N, Hashimoto K (2021). Comparison of salvage surgeries for lung adenocarcinoma treated with anaplastic lymphoma kinase-tyrosine kinase inhibitors. Current Problems in Cancer. Case Rep.

